# Worldwide Experiences in Disaster Medicine Education

**DOI:** 10.1017/dmp.2020.150

**Published:** 2020-05-07

**Authors:** Luca Ragazzoni, Monica Linty, Francesco Della Corte

**Affiliations:** CRIMEDIM – Research Center in Emergency and Disaster Medicine, Università del Piemonte Orientale, Novara, Italy

**Keywords:** disaster medicine, education, health education, public health professional

With this letter, we thank Professor Nobuyasu Komasawa and his colleagues for having highlighted in their letter to the editor^1^ the necessity to improve the collaboration in disaster medicine education and to strengthen the efforts in integrating this discipline in medical school curricula worldwide.

Just like Japan, Italy is widely exposed to natural disasters, and the scientific community is well aware that climate change will only exacerbate the situation and expose the country to new health threats.^2^ Despite the recognition and emphasis on the important role of medical professionals in disaster preparedness and response, medical and nursing students are still lacking basic education in disaster medicine,^3^ in Italy and worldwide. The Research Center in Emergency and Disaster Medicine (CRIMEDIM; Novara, Italy) has been working since early 2000 in trying to fill this gap, by fighting to include disaster medicine in the basic curriculum of medical students and implementing specific education programs on disaster medicine.

In 2013, a nationwide peer-assisted learning (PAL) program in disaster medicine, called DisasterSISM was developed in collaboration with the Segretariato Italiano Studenti in Medicina (SISM), the Italian branch of the International Federation of Medical Students’ Associations (IFMSA). The aim of this program, which is still running today, is to equip medical students with the necessary expertise and competencies to effectively transfer this knowledge to other students. From 2013 to 2018, the program managed to reach 88% of the Italian medical schools.^4^ Driven by the success of this national PAL program and by the growing awareness that disaster medicine is poorly represented in medical students’ curriculum worldwide, CRIMEDIM implemented an international version of the DisasterSISM. The Training Disaster Medicine Trainers (TdmT) was developed in collaboration with the IFMSA with the aim to create a global network of disaster medicine student-teachers. After completing the program, students are supported by CRIMEDIM and their local branch of IFMSA in planning and delivering basic courses in disaster medicine across the globe.

Both these programs exploit the multiplier effect of PAL to maximize the results and reach an always growing number of universities in the world. However, this is not the only way CRIMEDIM works to enhance the international collaboration in disaster medicine education. Since 2000, the European Master in Disaster Medicine has equipped professionals from all around the world with specific skills and knowledge in disaster medicine.^5^ Through the European-funded project NO-FEAR, CRIMEDIM coordinates a pan-European and beyond network of 18 partners among emergency medical care practitioners, academia, suppliers, decision-makers, and policy-makers to collaborate and exchange knowledge, good practices, and lessons learned in emergency and disaster management. Advancing knowledge and practice in education and training of medical practitioners is one of the main objectives of the project.

CRIMEDIM experience can be seen as a successful example of how academia can strengthen the presence of disaster medicine education in the undergraduate and postgraduate path and can have an important role in overcoming the fragmentation and filling the education gap pointed out by Komasawa and his colleagues. We strongly hope and believe that sharing examples of good practice could encourage the development of similar initiatives in other countries and enhance the international collaboration toward a common aim.
